# 3D Printing of Multimaterial Contact Lenses

**DOI:** 10.1021/acsbiomaterials.3c00175

**Published:** 2023-06-26

**Authors:** Muhammed Hisham, Ahmed E. Salih, Haider Butt

**Affiliations:** Department of Mechanical Engineering, Khalifa University, Abu Dhabi 127788, United Arab Emirates

**Keywords:** multimaterial 3D printing, vat photopolymerization, hydrogel, contact lenses, wavelength filtering

## Abstract

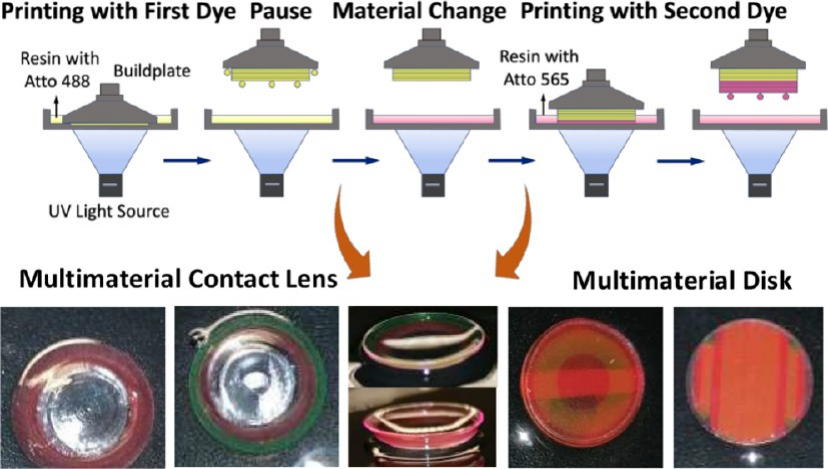

3D printing of multimaterial objects is an emerging field with promising applications. The layer-by-layer material addition technique used in 3D printing enables incorporation of distinct functionalized materials into the specialized devices. However, very few studies have been performed on the usage of multimaterial 3D printing for printable photonic and wearable devices. Here, we employ vat photopolymerization-based 3D printing to produce multimaterial contact lenses, offering enhanced multiband optical filtration, which can be valuable for tackling ocular conditions such as color blindness. A combination of hydroxyethyl methacrylate (HEMA) and polyethylene glycol diacrylate (PEGDA) was used as the base hydrogel for 3D printing. Atto565 and Atto488 dyes were added to the hydrogel for wavelength filtering, each dye suitable for a different type of color blindness. Multimaterial disks and contact lenses, with separate sections containing distinct dyes, were 3D-printed, and their optical properties were studied. The characteristics of multimaterial printing were analyzed, focusing on the formation of a uniform multimaterial interface. In addition, a novel technique was developed for printing multiple dyed materials in complex lateral geometrical patterns, by employing suitable variations in CAD models and the UV curing time. It was observed that the multimaterial printing process does not negatively affect the optical properties of the contact lenses. The printed multimaterial contact lenses offered a combined multi-band color blindness correction due to the two dyes used. The resulting optical spectrum was a close match to the commercially available color blindness correction glasses.

## 1 Introduction

Multimaterial 3D printing is a promising technology that has recently gained a myriad of research interest as it enables the production of 3D-printed objects with controlled variations in material composition and properties. Such multimaterial objects are beneficial in specialized applications where material and property variations across the object geometry provides interesting functionalities. Applications include producing biomimetic components, tissue engineering, controlled drug delivery, soft robotic components, and optical applications.^1−7^ Multimaterial 3D printing can significantly reduce production time and simplify the production process steps. The entire geometry (consisting of multiple sections of various materials) is printed in a single go, unlike conventional production processes where each part of a different material must be produced separately and assembled. Multimaterial printing is possible through various 3D printing methods, such as fused deposition modeling (FDM), material extrusion, and vat photopolymerization. Digital light processing (DLP) is a type of vat photopolymerization process where UV light is projected in required 2D patterns on the vat, using a suitable light projection technique. The DLP process is of great interest because of its high resolution and capability for printing hydrogels which are useful in biomedical applications.^8,9^ In addition, DLP is the most suited printing process for printing optical components as it provides smooth surface finish and low scattering losses in the printed samples.^10−14^ Strong and smooth bonding between the various printed layers (in DLP printing) also prevents significant optical transmission losses at the interfaces between the layers.

**Figure 1:**
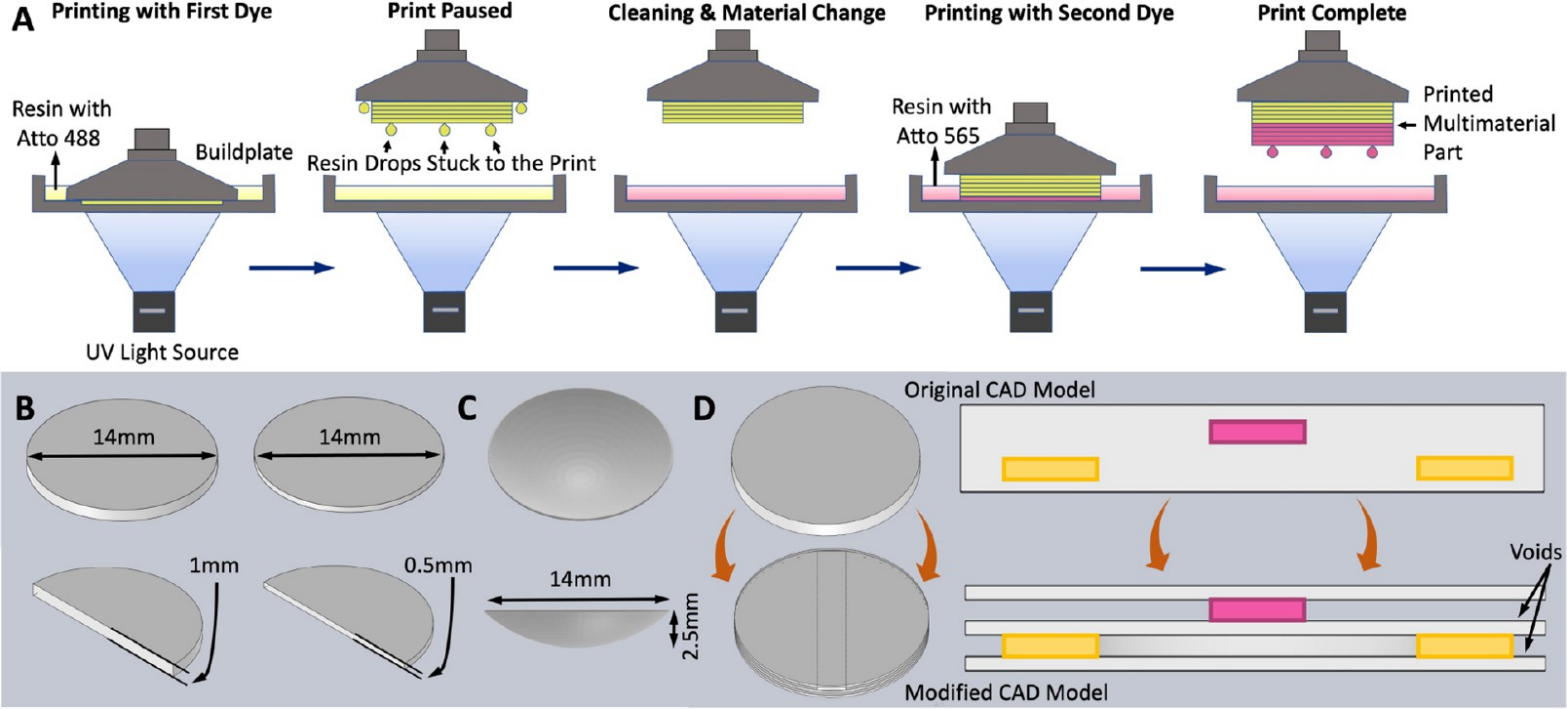
(a) Multimaterial 3D printing process used for producing discs and contact lenses with multiple embedded dyes achieving multiband optical filtering properties. (b) CAD models of optical disks used in this study, with 14 mm diameters and thicknesses of 1 mm and 0.5 mm. (c) CAD models used for printing single and multimaterial contact lenses. (d) CAD model modification for printing complex multimaterial patterns.

Several studies have explored the production of multimaterial parts with the DLP printing process.^15−19^ However, multimaterial printing requires certain modifications in the conventional DLP technique. In the DLP process, UV light is illuminated onto a vat (or tank) filled with a liquid photopolymer placed inside the printer. UV light causes the liquid photopolymer to cross-link (polymerize) and convert into a solid. This action is repeated layer-by-layer by projecting different UV light patterns into the resin bath, coupled with a step-by-step upward movement of the build plate, to finally produce the required 3D object.^9,20^ For multimaterial printing, the liquid photopolymer, inside the vat, is replaced whenever a material change is required.^21^ Modified DLP printers have been developed to enable automated material change. These printers achieve the change in material by either using an array of vats,^19,22,23^ or with partitioned material sections within a single vat,^24,25^ or by pumping mechanisms to change the materials in the vat,^26,27^ or by dropping puddles of material onto the vat which is cleaned by an air jet.^17^ However, DLP printers with these kinds of modifications are not widely available on a commercial scale. A more straightforward method for producing multimaterial parts with commercially available DLP printers is to pause the printer when required and manually change the material in the vat (Figure 1a). This method has also been used in some studies as it is economical and feasible, although a manual intervention is necessary.^15,28^

Studies on using multimaterial 3D printing of optical applications (such as multimaterial and functionalized contact lenses) have been rather limited. Joralmon et al.^19^ used multimaterial DLP printing to produce liquid crystals that change optical properties with temperature. Li et al.^29^ printed bilayer hydrogels that change color, brightness, and shape with pH. Iezzi et al.^30^ printed multimaterial 1D photonic crystals using an e-jet printing. Although the DLP process is very suited for optical components, there are certain challenges associated with multimaterial DLP printing that can limit the accuracy and properties of the printed samples. These issues also limit the production of multimaterial objects with complex patterns. These challenges include the formation of an interface at the point of a material change,^17^ the change in lateral dimensions with material change,^28^ and difficulty in producing variations in the lateral (*x*–*y*) plane.^24,25^ Previous studies have shown that lateral deviations can be resolved by suitable CAD model correction.^28^ However, there are no studies which sufficiently address the other two challenges. Rank et al.^25^ described the printing of samples with four different materials on the x-y plane by dividing the vat into four separate sections. However, this technique is applicable only for certain limited geometries and not applicable for more complex shapes, such as contact lenses.

In this work, we explored 3D printing of multimaterial contact lenses made up of two different dyes, each suitable for correcting a particular type of color blindness. A combination of hydroxyethyl methacrylate (HEMA) and polyethylene glycol diacrylate (PEGDA) was used as the liquid photopolymer solution for dissolving the dyes. Well known color blindness correction dyes, Atto565 and Atto488, were then added to this resin before printing. On printing, a tinted hydrogel based material is produced, which has properties suitable for use as contact lenses and wearable glasses. We explored the effect of the multimaterial printing process on the optical properties of these printed hydrogels, mainly focusing on the optical filtering exhibited by the multimaterial interface. In addition, we also demonstrate the novel printing of complex patterns, enabling material variations along the lateral plane. This technique successfully employs the effect of over curing (which is usually considered a limitation^31,32^) for facilitating lateral variations in the contact lens material composition. Our technique has the advantage of being compatible with a variety of sample shapes and pattern designs; however, certain modifications in the CAD model and curing time are necessary.

We focused on color blindness as an application as it is a vision deficiency that affects a large section of global population. Estimations show that it affects one in 12 males and one in 200 females.^33^ Previous studies have demonstrated the usage of 3D-printed contact lenses and wearable glasses for color blindness correction.^10,11,34−38^ There are three types of cone cells or photoreceptor cells in the human eye: S-cone (blue), M-cone (green), and L-cone (red).^39,40^ In the eyes of patients suffering from color blindness, one or more types of cone cells are either absent or malfunctioning. When S-cone malfunctions (with displaced activation wavelengths), the condition is called tritanomaly; when M-cone malfunctions, it is called a deuteranomaly; and when L-cone malfunctions, then it is called a protanomaly.^41−43^ Protanomaly and deuteranomaly are collectively known as red-green color blindness as the patients have difficulty distinguishing these two colors. Tritanomaly patients, on the other hand, have difficulty distinguishing blue and yellow colors. Hence, tritanomaly is also known as blue–yellow color blindness. These two types of color blindness can be corrected using lenses/glasses that filter suitable wavelengths for each type.^44,45^ Some patients may have more than one type of color blindness simultaneously. These patients require filtering of multiple wavelengths, one for each type of color blindness.

Salih et al.^34−36^ used gold and silver nanoparticles for producing nanocomposite color blindness correction contact lenses. Gold nanoparticles of sizes 12 and 40 nm were found to be suitable for red–green color blindness correction. Silver nanoparticles of size 60 nm were found to be suitable for blue–yellow color blindness correction. Roostaei et al.^38^ used gold nanolayer deposited on a 2D plasmonic structure for producing red–green color blindness correcting lenses. However, in the above two studies, the nanomaterial preparation and contact lens production strategy involved relatively complex and expensive procedures. 3D printing was not found to be feasible with the prepared nanoparticles. Sekar et al.^46^ found that silicone hydrogels containing natural woad and paprika pigments can help in color vision correction. Hittini et al.^37^ recently 3D-printed contact lenses with a low-cost ink which showed wavelength filtering suitable for red–green color blindness correction. Although these 3D-printed devices have not yet attained the efficacy of commercially available lenses and glasses, there is hope that 3D printing, with its novel abilities, will facilitate the production of highly customized vision aids capable of tackling color blindness as per every patient’s individual needs. The current study is hence a step toward that aim. Here, we show that multimaterial 3D printing can provide the advantage of using multiple dyes within one sample, thus providing the ability to correct more than one type of color blindness. This work demonstrates the use of multimaterial printing to simultaneously correct both red–green and blue–yellow color blindness by filtering two suitable optical wavelength bands. The printed multimaterial hydrogels could display a transmission and absorption spectrum close to commercial glasses Enchroma and BJ-5149. 3D-printed lenses and glasses have the advantage that they can be easily customized to suit the specific needs of a patient, whereas commercial devices are not easily customizable. Furthermore, the multimaterial process enables controlled deposition of dye at required locations within the lens geometry, which can be very useful for producing multifunctional contact lenses in the future.

## 2 Materials and Methods

### 2.1 Materials

For the preparation of the resin, HEMA was used as the monomer, PEGDA served as the cross-linker, and trimethyl benzoyl diphenylphosphine oxide (TPO) served as the photoinitiator. For washing 3D-printed samples, isopropyl alcohol (IPA) was used. The hydrogel was colored using Atto565 and Atto488, two fluorescent dyes. Dimethyl sulfoxide (DMSO), obtained from Merck chemicals, was used as a solvent to dissolve the dyes. All other compounds were purchased from Sigma-Aldrich.

### 2.2 Resin Preparation

For 3D printing, a UV-curable liquid photopolymer resin that consists of HEMA and PEGDA is used. Hydrogels made of PEGDA alone are brittle and crack easily during swelling/shrinkage.^28^ Hence, HEMA was added as a monomer to make the hydrogel more flexible and reduce the occurrence of crack formation. TPO is the UV-sensitive photoinitiator, which serves as an initiator to start the photopolymerization reaction. PEGDA is a long-chain, hydrophilic, cross-linking monomer that reacts when initiated by TPO to form the solid hydrogel network. To form the resin, HEMA and PEGDA are mixed in a ratio of 1:1 by volume, and TPO is added to the solution at 5% by weight, all while the mixture is continually stirred. The liquid resin is then suitably mixed with wavelength-filtering dyes (Atto565 and Atto488). First, 1 mg of the dye is dissolved in 1 mL of DMSO to produce 1 mL of liquid dye. Then, the dye is added in suitable proportions to the hydrogel resin, previously prepared and mixed thoroughly. Three different concentrations of dye were used in this study: 1.25, 2.5, and 5% by volume. The different concentrations of each dye were categorized for identification based on the dye’s type and volume percentage. For example, Atto488/1.25% denotes the resin containing Atto488 at 1.25% (volume of liquid dye: volume of resin). Also, solutions containing the Atto565 and Atto488 dyes were produced by mixing the prepared Atto565 and Atto488 containing resins in a ratio of 1:1 by volume.

### 2.3 CAD Modeling

The CAD software SOLIDWORKS is used to create the designs for 3D printing. The file is saved in standard triangle language (.stl) format because this is the preferred input for all slicing tools. A simple disk-shaped model (diameter: 14 mm, thickness: 1 mm or 0.5 mm) was used for testing the multimaterial printing and the resulting optical, mechanical, and hydration properties (Figure 1b). A contact lens CAD model (14 mm diameter, 2.5 mm depth, and 200 μm wall thickness) was used for printing multimaterial contact lenses (Figure 1c). For printing multimaterial disks with complex patterns, modified CAD models were prepared. Here, each section of a different material is modeled as a subsequent section along the *z*-axis, leaving voids where intra-layer material change is required (Figure 1d). This is done so that a very high curing time can be used while printing to compel material formation on the subsequent section and to fill the voids. This process is discussed in more detail in the Section 3.

### 2.4 3D Printing

A DLP-based 3D printer (Wanhao D8) was used for printing. The printer uses a 405 nm UV LED and a DLP projector. The .stl file that was prepared using SOLIDWORKS was transformed using a slicing tool (Chitubox slicer) into printer-readable .zip format containing the projected images and print instructions as the G-code. As shown in Table 1, the print settings have been optimized for the current resin and printer. However, for printing complex designs, a cure time of 100 s was used at specific points to make use of the overcure effect. The resin vat was filled with the prepared hydrogel resin, and the printing was done by transferring the prepared file to the printer. For printing multimaterial samples, the printer was paused at the required time steps. Pausing causes the printing process to stop, and the build plate rises to allow manual access to the printed sample. The liquid resin in the vat was removed manually and replaced by a different resin (with a different dye). The printed sample sticking onto the build plate was also cleaned to remove traces of resin sticking onto it. Cleaning was performed to prevent mixing of previously used material with the new material poured into the vat. The sample was cleaned very carefully, as rough handling can cause it to detach from the build plate. It was cleaned by running IPA over it and then wiped dry. The printing process was then resumed, thus causing the sample to be printed with two dyed resins. The printed sample was removed from the build plate and sonicated for 20 min while immersed in IPA. The sonication process removes traces of uncured resin still attached to the sample, making the surfaces smooth and clean. Thorough sonication after printing greatly improved the sample’s optical transmission, which is crucial for optical applications and contact lenses.

**Table 1:** Parameters for 3D Printing that Were Used To Print the Hydrogel Disks

printing parameters	specifications
layer thickness	35 μm
curing time	burn layers −80 s, normal layers −25 s
burn layer count	5 layers
lift distance	6 mm
lift speed	50 mm/min
retract speed	50 mm/min

### 2.5 Characterization

The optical transmission/absorption, water absorption capacity, and dye leakage of the produced tinted sample were characterized. The transmission and absorption spectra of the liquid resin and 3D-printed samples were measured using an Ocean Optics UV–vis spectrophotometer (USB 2000+, by Ocean Optics), which has a detection range of 400–1100 nm. Plots are made showing the transmission intensity (%) vs wavelength (nm) and absorption (optical density (OD)) vs wavelength (nm). Images of the sample were used for comparing the distinct tints of different dye combinations. Cross-sections of the sample were photographed to compare the standard and multimaterial printing. Samples were cross-sectioned by breaking them manually after drying, causing them to fracture in a brittle manner. The cross-sections were imaged using a ZEISS Axiocam 105 color camera attached to a ZIESS Axio Scope A1 optical microscope (at 5× magnification). By soaking the samples in water and weighing them at periodic intervals, the water absorption capacity of these 3D-printed hydrogel samples was evaluated. Water absorption and retention is a crucial parameter for hydrogel-based soft contact lenses. All samples were dried in an oven at 60 °C for 1 h. The weight of samples was measured, initially in the dry state and later after they were immersed in water for various amounts of time. The difference in weights was used to determine how much water had been absorbed. The water absorption capacity (%) is calculated using equation 1.
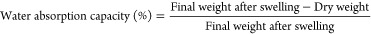
1

For the proper functioning of the tinted contact lenses (as optical filters), it is imperative that the dye should not leak from the 3D-printed hydrogel media. In addition, the two dyes used in the multimaterial lenses should not leak across the interfaces and intermix. The dye leakage was studied by measuring the lens’ absorption spectrum at different intervals, after immersion of the sample in water. Measurements were done at intervals: 1 day, 1 week, and 1 month. The intensity of the absorption peak at different periods was compared with the absorption peak in the dry state to observe if there is any loss in intensity due to dye leakage. Additionally, images of the sample cross-section are taken at these intervals and compared.

To study the structural artifacts at the multimaterial interface, very thin samples were printed with the following combinations and imaged under an optical microscope. First, 2 mm wide samples were printed using clear resin (without any dye) with cure times of 20, 25, and 50 s. These samples were printed in three forms: continuous print, print paused for 5 min (without cleaning or material change), and print paused for 5 min and cleaned (without material change). Second, 2 and 10 mm wide samples were printed with a cure time of 25 s, with the following multimaterial combinations: clear:clear (only pausing and cleaning), clear:Atto565, Atto565:Atto488, and Atto488:clear. The multimaterial-layered interface was imaged under an optical microscope. These images were compared to understand the characteristics of the interface that forms when the material change occurs.

## 3 Results and Discussion

**Figure 2:**
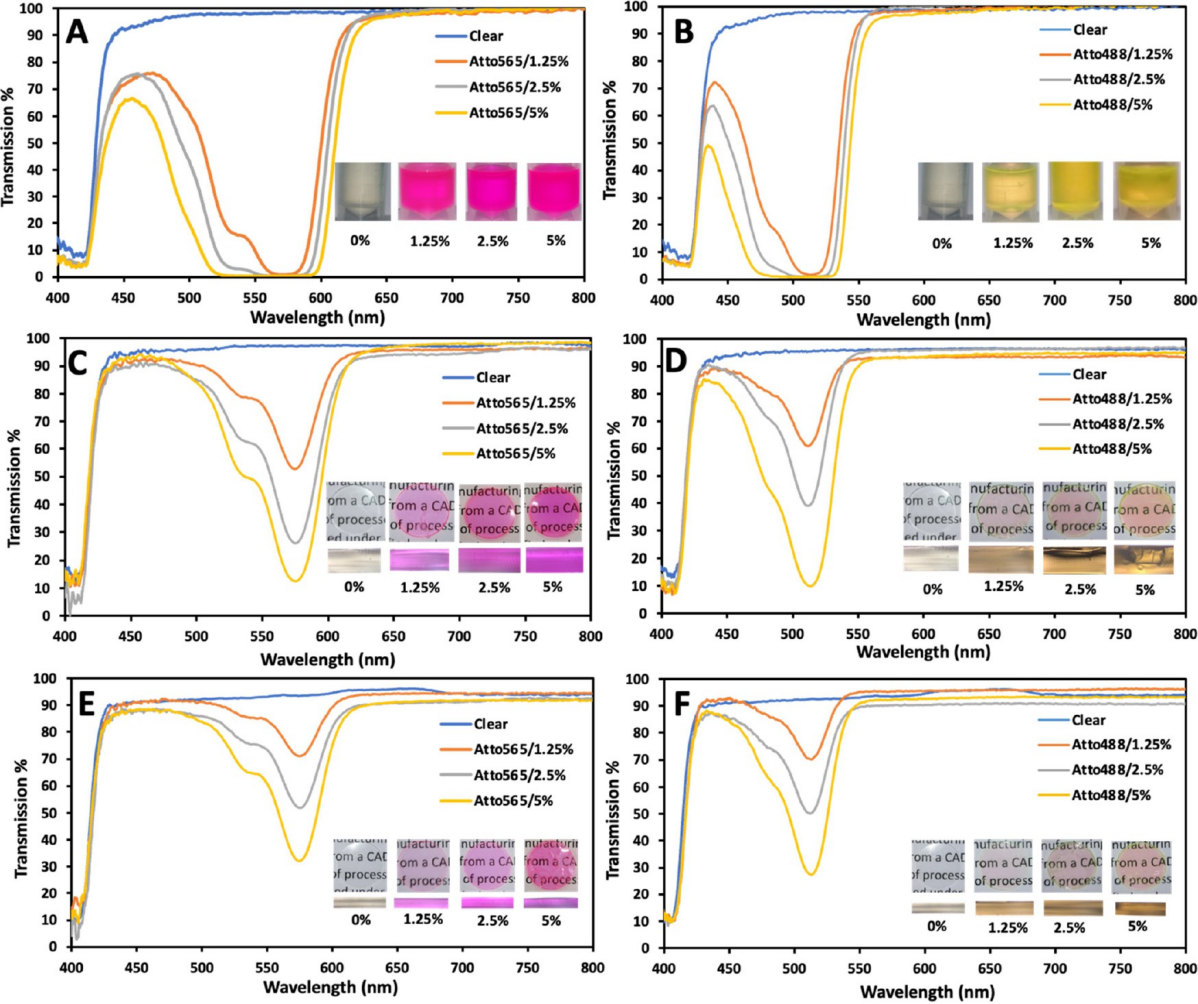
Transmission spectra from liquid resin tinted with (a) Atto565 and (b) Atto488 dyes. Inset shows the images of the liquid resin. Transmission spectra of 3D-printed disks with (c) Atto565 dye and 1 mm thickness; (d) Atto488 dye and 1 mm thickness; (e) Atto565 and 0.5 mm thickness; and (f) Atto488 dye and 0.5 mm thickness. The inset shows the surface and cross-sectional images of the 3D-printed samples.

Atto565 dye is visibly pink, whereas Atto488 dye is yellow–green. When these dyes were dissolved in DMSO, they formed very stable solutions. Furthermore, a stable solution was formed when the DMSO-dye solution was mixed with the HEMA/PEGDA resin. After the initial mixing, the dye did not separate from the resin. Transmission results for the dyed resins show transmission dips around 572 nm for Atto565 and around 513 nm for Atto488 (Figure 2a,b). Furthermore, another sharp dip occurs around 370–420 nm, due to the presence of TPO in the resin, which absorbs UV light in this range. This same dip at 370–420 nm also occurs for clear resin without the dye, as the TPO content is the same in clear resin and resin with the dye. The transmission dip increases with an increase (1.25 to 5%) in the concentration of the dye used. The corresponding absorption spectrum is given in the Supporting Information (Figure S1). The absorption spectra display peaks at the same wavelength, where a dip occurs in the transmission mode measurements (Figure 2). The intensity and width of absorption peaks increases with the dye concentration. In addition, it was observed that the transmission at other wavelengths (outside the filtered bands) was very high (around 90% or higher). The high transmission shows that highly transparent optical components (contact lenses) can be 3D-printed using our method, provided surface cleaning through sonication is performed post-printing. Again, the dip’s intensity and width increase with an increase in dye concentration.

The intensity and the widths of the dips were observed to depend on the thickness of the 3D-printed samples and on the dye concentration. The dip intensity and full width at half maximum (FWHM) both increase with an increase in sample thickness and an increase in dye concentration (Figure 2c). For samples with Atto565, the transmission dip occurred with an average dip intensity of 71% (for 1 mm thick samples at 1.25% dye concentration) and an average FWHM of 549–593 nm. Similarly, for Atto488 samples, the transmission dip had an average intensity of 61% (for 1 mm thick samples having 1.25% dye concentration) and an average FWHM of 482–527 nm. Molar absorptivity calculations based on the absorbance spectrum of 3D-printed samples showed relatively close maximum absorptivity values for both the dyes (Figure S4). Interestingly, the transmission from clear samples (printed using the clear resin) does not vary with the increasing thickness of the sample. It generally transmits over 90% of visible spectrum, irrespective of the thickness. The thickness-independent transmission shows that there is negligible loss in transmission due to the layer-by-layer printing process. It is visible from the cross-sectional images of the samples that the individual layers have bonded smoothly, leaving no defects, air bubbles, or layer boundaries. Hence, there is negligible scattering loss as light passes through the clear 3D-printed samples. The slight transmission loss occurs at sample surfaces, where minute distortions exist, producing a small loss in intensity due to back reflection.

**Figure 3:**
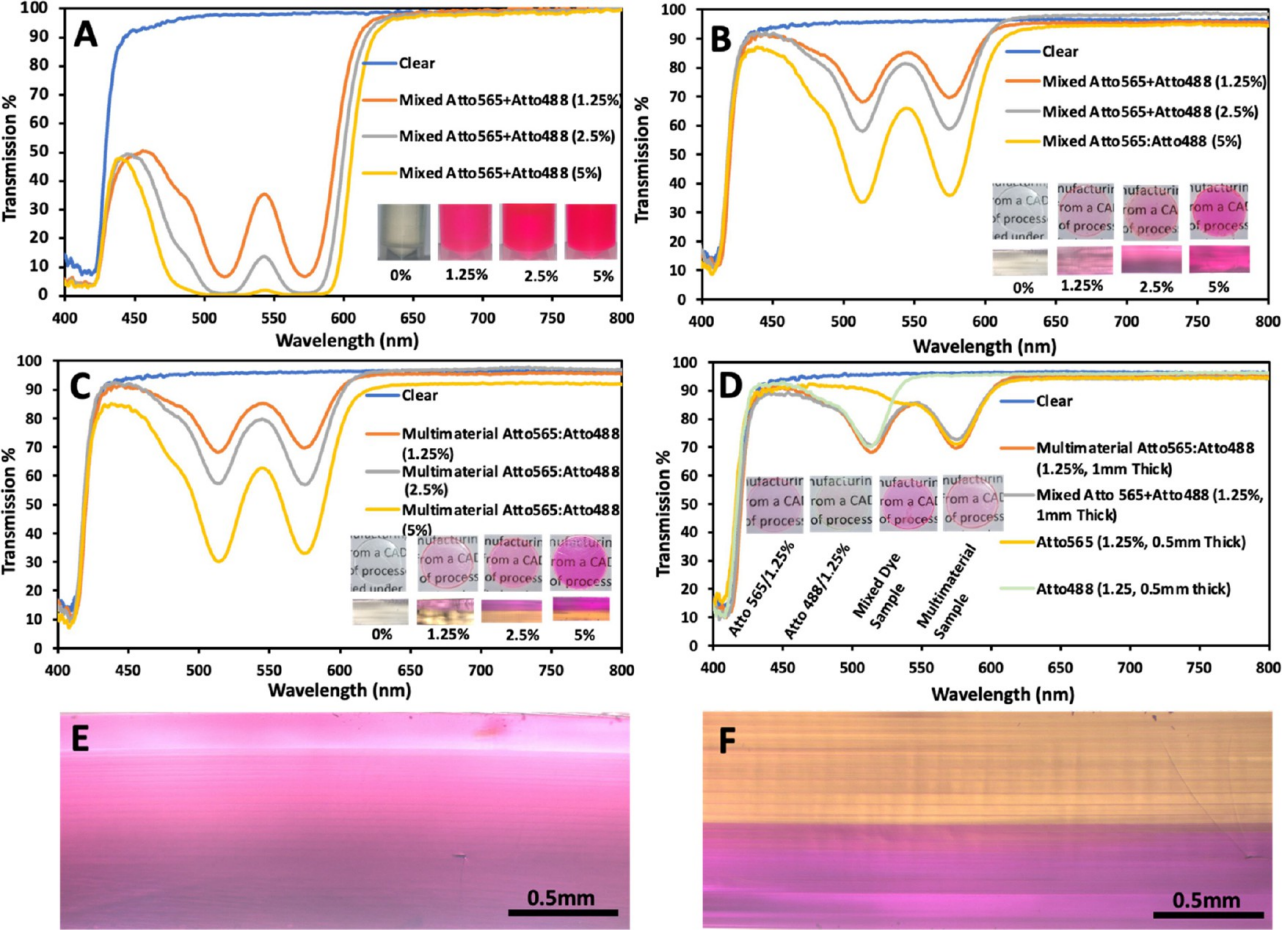
(a) Transmission spectra of liquid resin with both Atto565 and Atto488 mixed. Inset shows the images of liquid resins. Transmission spectra of 3D-printed disks (1 mm thick) made of (b) Atto565 and Atto488 mixed together, and (c) multimaterial samples having Atto565 and Atto488 in 0.5 mm thick separate sections. Inset shows the 3D-printed samples and the cross-sectional images. (d) Comparison of transmission spectra for multimaterial, mixed, and single composition samples of the same concentration (1.25%). The inset shows the 3D-printed samples. Cross-sections of printed disks made of (e) mixed Atto 565 + Atto 488 resin and (f) multimaterial Atto565 and Atto488, both of concentration 2.5%.

Mixing resins simultaneously with two dyes also produced a stable resin-dye formulation. The transmission from mixed Atto565 + Atto488 shows the presence of two transmission dips, each corresponding to the original dip from each dye (Figure 3a). The 3D-printed samples produced from the mixed dyed resin also showed similar transmission behavior with two dominant dips, one from each dye (Figure 3b). Interestingly, the 3D-printed multimaterial samples also showed the same behavior (Figure 3c). These samples (1 mm thick) had a half section of 0.5 mm thickness printed with Atto565 and another half of 0.5 mm thickness printed with Atto488 dye resin. The intensity of the dip and their FWHM were identical for the mixed and multimaterial samples, for their respective dye concentrations. The only difference was that when cross-sectioned, the mixed dye sample showed a uniform color corresponding to the mixed color of both dyes. In contrast, the multimaterial sample showed two separate sections showing the pink and yellow colors of the respective dyes. The transmission dips displayed by the mixed and multimaterial samples also matched with the optical response of the 0.5 mm thick samples printed with individual dyes (Figure 3d). This match in dip intensities occurred because each multimaterial sample had one Atto565 section of 0.5 mm thickness and one Atto488 section of 0.5 mm thickness, while the mixed samples had the same net amount of dye in the sample (but distributed uniformly within the printed volume). These results show that Atto565 and Atto488 dyes do not react with each other when mixed. Hence, they retain their optical properties even after mixing. Also, the results show that the multimaterial printing process does not affect the optical properties. The transmission intensity and the colors were the same with standard and multimaterial printing. Previous studies have reported the formation of multimaterial interfaces/boundaries on the printed sample surfaces.^17^ Our printed multimaterial geometries also had a small interface on the external most circular surface. However, it was observed that the cross-section of the multimaterial sample does not show any such interface inside it. Internally, the two phases have smoothly bonded without the presence of any discontinuities that may affect the optical transmission. Hence, multimaterial printing can be successfully utilized to produce optical devices which employ multiple dyes or other composite resins. Particularly in situations where the dyes react with each other when mixed directly, multimaterial printing can produce samples with both dyes without direct mixing.

**Figure 4:**
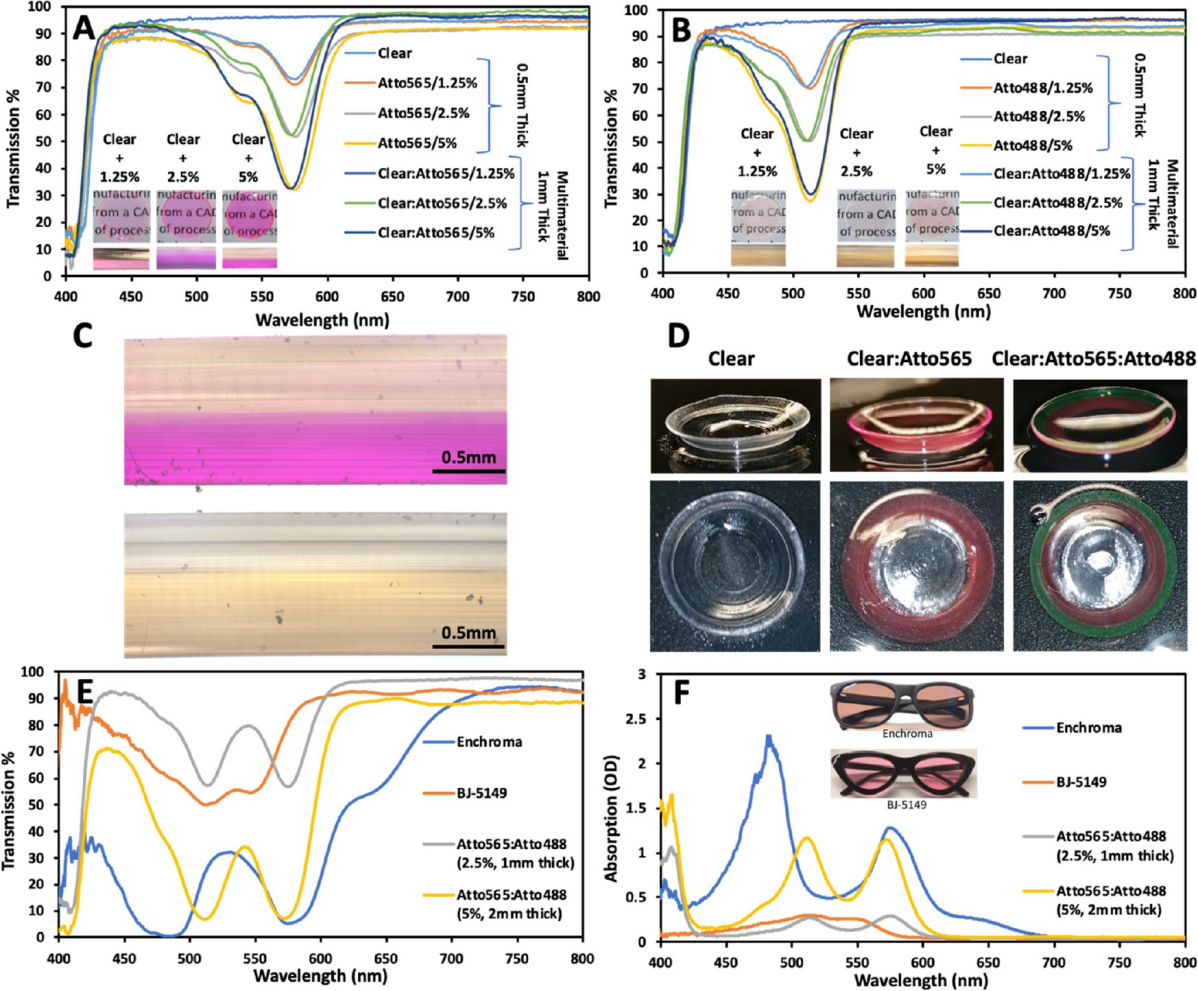
Comparison of transmission spectra from 3D-printed single-material disks (thickness 0.5 mm) with multimaterial disks (thickness 1 mm) having combination (a) clear:Atto565 and (b) clear:Atto488. (c) Cross-sections of clear:Atto565 and clear:Atto488 disks having concentration 2.5%. (d) Multimaterial contact lenses printed with clear resin and resins containing Atto565 and Atto488. (e) Transmission spectra and (f) absorption spectra of commercial color blindness glasses compared with 3D-printed multimaterial samples.

Multimaterial samples printed with a combination of clear and single dyed resin (clear:Atto565 or clear:Atto488) displayed a transmission spectrum with a single band of optical filtration as if the section made of clear resin is fully transparent (Figure 4a,b,c). These samples had a 0.5 mm thick section printed with a clear resin and another 0.5 mm thick section containing the respective dye. The transmission dip from these samples matched the dip obtained from the 0.5 mm thick single-material samples, made of the respective dye and concentration. The matching dips again confirm that the multimaterial printing does not alter the optical behavior of the samples. Multimaterial contact lenses were also 3D-printed using the same printing technique (Figure 4d). Contact lenses were printed with clear resins, clear + Atto565 dyed resins, and with clear + Atto565 + Atto488 dyed resin combinations. The central region were clear with Atto dyes present in the subsequent curved rings of the lenses. The printed samples demonstrate that a variety of combinations can be used to print multimaterial contact lenses for optical filtering and other functional applications.

Comparing the transmission and absorption spectrum of multimaterial samples with the spectra of commercially available color blindness correction glasses shows closely similar spectral behavior, much closer agreement than that was previously possible whilst using individual dyes^11^ (Figure 4e,f). Enchroma has transmission dips around 486 and 575 nm with respective intensities of 0 and 5%. This is close to the spectra obtained for Atto565:Atto488 (5%, 2 mm thick), which has transmission dips around 513 nm (7%) and 572 nm (7%) and FWHM 468–596 nm. Also, BJ-5149 has dips around 513 and 546 nm with respective intensities of 50% and 55%, and FWHM 450–570 nm. This is close to the spectra obtained for Atto565:Atto488 (2.5%, 1 mm thick) with dips 513 nm (57%, FWHM 489–539 nm) and 572 nm (57%, FWHM 550–593 nm).

**Figure 5:**
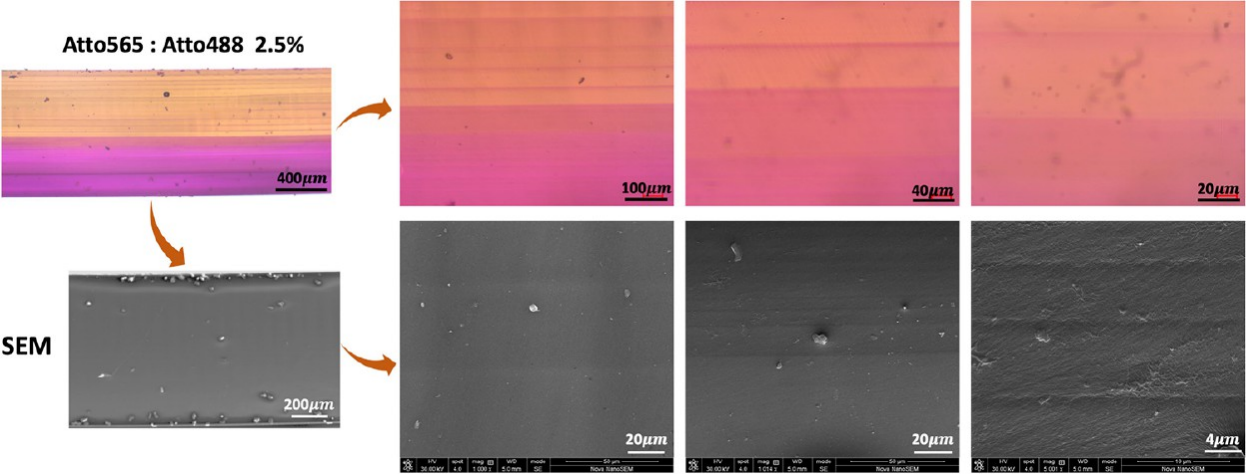
Highly magnified images of the cross-section of multimaterial disk obtained using optical microscopy and SEM.

As mentioned previously, no discontinuities are visible inside the sample to indicate a material change interface. Magnified images of the region where material change occurs show the same, but a small region where the two dyes have mixed was also visible (Figure 5). This mixing region, consisting of a few layers, was visible in a distinct tint, which was produced when both the dyes were mixed. Mixing of the two materials is a challenge that is difficult to overcome completely while printing. Even with a thorough cleaning step during the material change, some small amount of mixing still occurs. When cleaning is not appropriately performed, the mixing effect can get even worse, leaving the latter printed section with dispersions of the first material. SEM images of the cross-sectioned sample also do not indicate the presence of any physical grain boundary or interface between the two printed materials (Figure 5). The SEM images show the whole sample in the same color as the SEM microscope (is not sensitive to color changes) depicting surface uniformity. A few lines are visible on the cross-section surface, but these lines are thought to form due to the brittle fracture that occurs when the sample is cross-sectioned.

**Figure 6:**
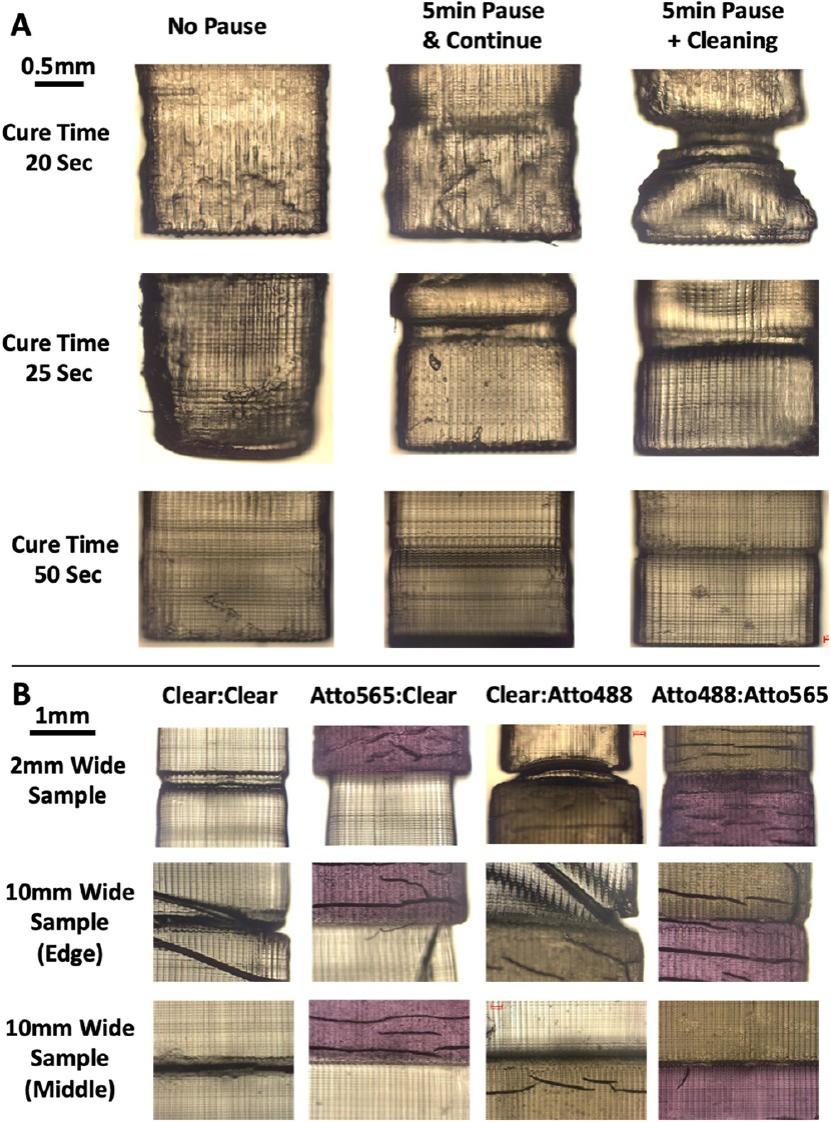
Multimaterial interface boundary, visible on the outermost surfaces of the 3D-printed samples. (a) Multimaterial interface boundary for same material (clear resin) under different cure times and pause conditions. (b) Boundary effect for multimaterial printed samples at cure time 25 s and with pausing, cleaning, and material change in between.

Although no boundary is visible inside the sample, a multimaterial grain boundary is visible on the outer most surface edge of the samples. This boundary (interface) was further studied with the help of small-sized samples (width = 2 mm) and larger depths (2 mm before the multimaterial grain boundary and 2 mm after the boundary). This shape enables proper viewing of this boundary under an optical microscope (Figure 6). Surprisingly, the results show that this interface forms on pausing the print job, without any material change. For clear:clear samples, a boundary forms with a 5 min pause at all cure times (Figure 6a). Cleaning appears to increase the severity of the discontinuous interface slightly. With an increase in cure time, the severity of the discontinuity appears to decrease. Additionally, the general appearance of the surface also improves at higher cure times, with the small discontinuities mostly disappearing. Only the ordered vertical lines that occur due to the pixel distribution of the printer’s projector remain as discontinuities. The multimaterial samples also showed a similar discontinuous interface just like the clear:clear samples (Figure 6b). However, the interface’s discontinuity is worsened because different sections (of different materials) have slightly different widths when printed. The printed section with Atto565 and Atto488 dye resin is wider than the section with clear resin. In the 10 mm wide sample, the difference in width between sections was still visible but comparatively less apparent. Previous studies have shown that the change in the lateral dimension that occurs with material change is a constant change at the edges of the sample, independent of sample dimension.^28^ Thus, the same difference in width would occur at the edges for 2 and 10 mm wide samples, which makes the difference less apparent in 10 mm wide samples. Although an interface discontinuity is still present in the central portion of the 10 mm wide samples, it appears more uniform.

**Figure 7:**
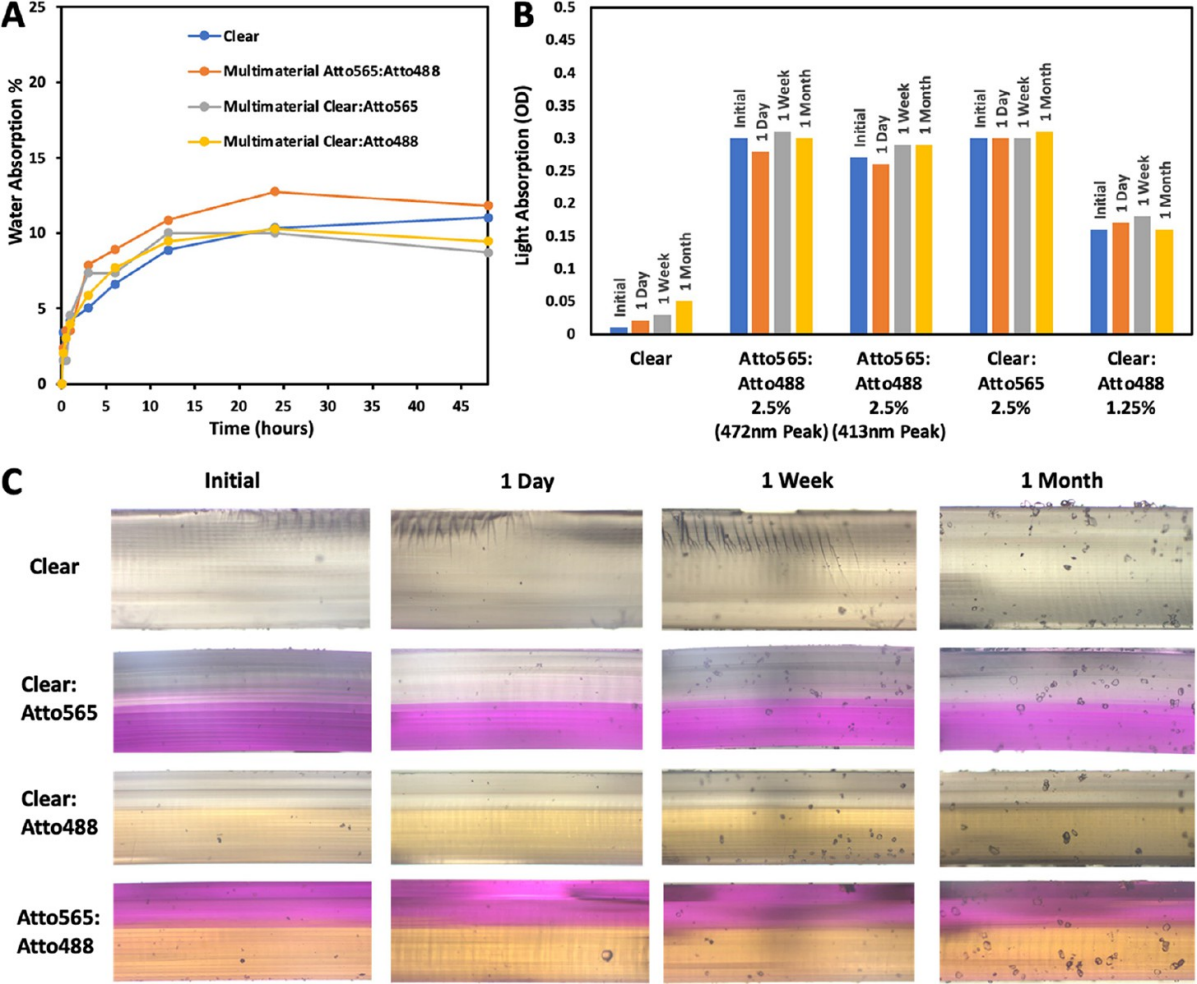
(a) Water absorption of dry 3D-printed samples, immersed in DI water. (b) Light absorption peaks of multimaterial samples immersed in DI water for extended periods for studying dye leakage and (c) images of respective samples.

Swelling studies (Figure 7a) showed that water absorption capacity is around 10% for all samples (with only slight deviations around this value, 1–2%). The samples took approximately 24 h to reach a completely swollen state from a dry state. There was no significant difference between single-material and multimaterial samples. Thus, multimaterial printing does not affect water absorption capacity. Although the maximum water absorption of the printed samples was relatively low, it can be increased significantly by increasing the HEMA concentration in the hydrogel resin (Figure S5). Studies on dye leakage indicated that the two dyes are completely stable inside the printed samples, with no leakage visible (Figure 7b,c). The optical spectrum from multimaterial samples showed that the spectrum does not change with time in DI water, and the intensity of the absorption peak was constant. The images of cross-sections (immersed in DI water) at various periods show the same color. For multimaterial samples, the two sections’ colors remained separate without mixing. However, small pits start appearing on the sample’s surface after an extended period. These tiny pits form due to the slow degradation of the printed hydrogel. A very small increase in absorption occurs (particularly apparent in the clear sample) due to this degradation. Surface analysis of multimaterial contact lenses revealed the staircase effect produced due to the layer-by-layer material addition during 3D printing (Figure S6). However, this effect can be significantly reduced by post-processing steps as reported in previous studies.^47,48^ Mechanical testing was performed to determine the tensile properties of 3D-printed hydrogel samples (Figure S7). The tensile modulus for these samples was somewhat higher than commercial soft contact lenses. This is apparently due to the large amount of PEGDA present in the hydrogel, which is known to increase the mechanical strength. The tensile modulus can be easily reduced by increasing the concentration of HEMA in the hydrogel. Moreover, cytotoxicity of the 3D-printed contact lenses was evaluated by measuring the cell viability of human dermal fibroblasts in hydrogel samples after 24 h. The 3D-printed HEMA/PEGDA hydrogels were found to be noncytotoxic as their viability was more than 85% (Figure S8).

**Figure 8:**
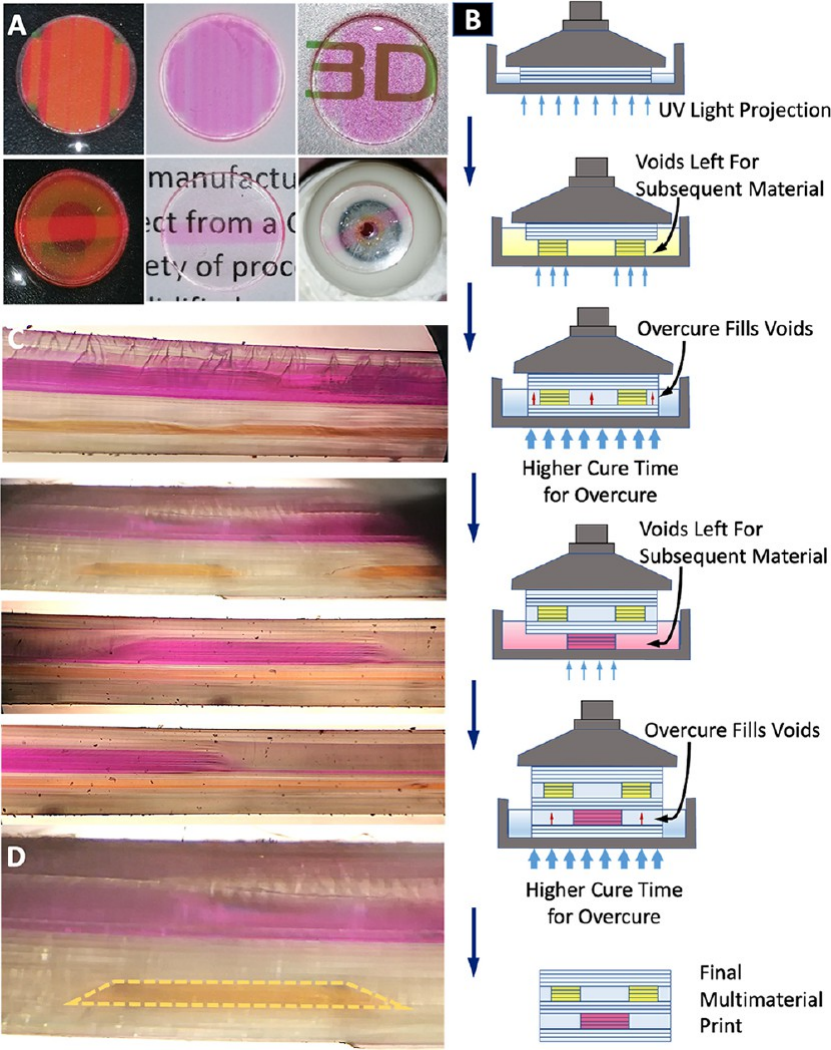
(a) Multimaterial disks (made of clear, Atto565, and Atto488 resins) printed in two complex patterns. (b) Process used to print these complex patterns. (c) Cross-sections of these patterned disks. (d) Cross-section showing Atto488 surrounded by clear resin on all sides, which is not possible to produce through the normal DLP printing process.

Finally, a novel technique for printing complex multimaterial patterns is shown in Figure 8. The technique works by modifying the CAD model, establishing different materials as separate sections along the *z*-axis. The regions where another subsequent material is needed are left as voids. While printing, a very high cure time (100 s in this case) is employed, at required points to compel the subsequent material to cross-link (and polymerize) within the voids left behind in the previously printed sections. Thus, the final print ends up as a continuous sample without voids but possessing variations in material along the *x*–*y* plane. Enabling the material change across the *x*–*y* plane (with such convenience) is a significant achievement for our method because in standard DLP multimaterial printing, only a material change across the *z*-axis is possible. Material change within the *x*–*y* plane is not possible in standard multimaterial printing because of the layer-by-layer printing scheme, which restricts material change to occur only on subsequent layers. It is impossible to return to a previously printed layer and deposit another material in it. However, here, we used suitable voids in initial layers and high cure times (in the following layers), due to which materials from subsequent layers reached back into initial layers through overcuring.

The samples printed with this technique are shown in Figure 8a, where specific patterns of Atto565 and Atto488 doped regions are printed within the clear sample. Regions where both the dyes overlap exhibit a variety of different tints. The yellow color (Atto488) was visible over a black background but was not easily distinguishable from the clear resin when kept on a white background. The cross-sections also show the complicated patterns of dye depositions within the 3D-printed samples. Figure 8d shows a region with Atto dye completely surrounded by clear resin on all sides. This kind of pattern is not possible in typical multimaterial prints. There is also great flexibility in the patterns that this novel technique can produce. The only requirement is a suitable modification of the CAD model and an increase in cure time (for overcuring to occur) at the required points. Some DLP printers allow the use of different cure times for different layers. However, this option is not possible for other DLP printers due to their software limitations. In these latter printers, this technique can still be implemented (with some limitations in the patterns possible) by using a very high cure time for all layers. However, in such cases, the print time can get very high here, which is undesirable. Overall, this study shows that DLP multimaterial printing has excellent capabilities for printing patterned samples without loss in optical properties. Thus, it provides tremendous opportunities for the future of optical and biomedical applications. The technique can be used for printing contact lenses with multi-filtering dyes combinations and the dyes can be printed in the central region of the lenses, so that the bulk of the lens remains transparent, or vice versa. It can also enable the easy production of multifunctional contact lenses in the future, by enabling the easy deposition of different dyes at the required location within the lens geometry. Current limitations for the process include the requirement for a manual intervention to the 3D printing process, difficulty for cleaning the sample at the point of material change, and certain limitations to the material distribution pattern. Future research can potentially address these limitations and further explore the 3D printing of multifunctional contact lenses by using dyes with various different functionalities located suitably within a contact lens.

## 4 Conclusions

Multimaterial disks and contact lenses containing Atto565 and Atto488 were successfully 3D-printed, providing an optical performance very similar to the commercially available color blindness correction glasses, a milestone not previously accomplished. There was no loss in optical transmission due to the multimaterial 3D printing. A grain boundary was visible on the outer surface of the samples; however, no significant discontinuity was visible inside the sample’s cross-section. The optical properties were the same whether the two dyes were directly mixed or printed separately in a multimaterial print. The similar optical properties for mixed and multimaterial samples indicate that the two Atto dyes do not chemically react with one another (or with the hydrogel media) when mixed and UV-cured. However, for other dyes that mutually react when mixed, multimaterial printing provides an alternate route to incorporate them into the same hydrogel sample. The multimaterial samples also displayed no leakage, and the color remained the same even after immersion in DI water for extended periods. Finally, complex multimaterial patterns were 3D-printed within the multimaterial contact lenses by using adequate CAD model modifications and controlled UV curing times. It is clear that multimaterial 3D printing is a feasible and valuable technique for the development of specialized optical and wearable devices.

## Supporting Information Available

Light absorption spectrum; surface characterization; mechanical properties; and cytotoxicity results (PDF)

## References

[ref1] Liu Z.; Meyers M. A.; Zhang Z.; Ritchie R. O. Functional gradients and heterogeneities in biological materials: Design principles, functions, and bioinspired applications. Prog. Mater. Sci. 2017, 88, 467–498. 10.1016/j.pmatsci.2017.04.013.

[ref2] Song Z.; Ren L.; Zhao C.; Liu H.; Yu Z.; Liu Q.; Ren L. Biomimetic Nonuniform, Dual-Stimuli Self-Morphing Enabled by Gradient Four-Dimensional Printing. ACS Appl. Mater. Interfaces 2020, 12, 6351–6361. 10.1021/acsami.9b17577. 31920076

[ref3] Missinne J.; Misseeuw L.; Liu X.; Salter P. S.; van Steenberge G.; Adesanya K.; van Vlierberghe S.; Booth M. J.; Dubruel P. Planar polymer waveguides with a graded-index profile resulting from intermixing of methacrylates in closed microchannels. Opt. Mater. 2018, 76, 210–215. 10.1016/j.optmat.2017.12.039.

[ref4] Salmoria G. V.; Klauss P.; Zepon K.; Kanis L. A.; Roesler C. R. M.; Vieira L. F. Development of functionally-graded reservoir of PCL/PG by selective laser sintering for drug delivery devices. Virtual Phys Prototyp. 2012, 7, 107–115. 10.1080/17452759.2012.687911.

[ref5] Jo H.; Yoon M.; Gajendiran M.; Kim K. Recent Strategies in Fabrication of Gradient Hydrogels for Tissue Engineering Applications. Macromol. Biosci. 2020, 20, 1900300 10.1002/mabi.201900300. 31886614

[ref6] Kuang X.; Wu J.; Chen K.; Zhao Z.; Ding Z.; Hu F.; Fang D.; Qi H. J. Grayscale digital light processing 3D printing for highly functionally graded materials. Sci. Adv. 2019, 5, eaav5790 10.1126/sciadv.aav5790. 31058222 PMC6499595

[ref7] Ker D. F. E.; Wang D.; Behn A. W.; Wang E. T. H.; Zhang X.; Zhou B. Y.; Mercado-Pagán Á. E.; Kim S.; Kleimeyer J.; Gharaibeh B.; Shanjani Y.; Nelson D.; Safran M.; Cheung E.; Campbell P.; Yang Y. P. Functionally Graded, Bone- and Tendon-Like Polyurethane for Rotator Cuff Repair. Adv. Funct. Mater. 2018, 28, 1707107 10.1002/adfm.201707107. 29785178 PMC5959293

[ref8] Li V. C.-F.; Kuang X.; Mulyadi A.; Hamel C. M.; Deng Y.; Qi H. J. 3D printed cellulose nanocrystal composites through digital light processing. Cellulose 2019, 26, 3973–3985. 10.1007/s10570-019-02353-9.

[ref9] Xue D.; Zhang J.; Wang Y.; Mei D. Digital Light Processing-Based 3D Printing of Cell-Seeding Hydrogel Scaffolds with Regionally Varied Stiffness. ACS Biomater. Sci. Eng. 2019, 5, 4825–4833. 10.1021/acsbiomaterials.9b00696. 33448825

[ref10] Alam F.; Salih A. E.; Elsherif M.; Butt H. Development of 3D-Printed Glasses for Color Vision Deficiency. Adv. Eng. Mater. 2022, 2200211 10.1002/adem.202200211.

[ref11] Alam F.; Salih A. E.; Elsherif M.; Yetisen A. K.; Butt H. 3D printed contact lenses for the management of color blindness. Addit. Manuf. 2022, 49, 102464 10.1016/j.addma.2021.102464.

[ref12] Vallejo-Melgarejo L. D.; Reifenberger R. G.; Newell B. A.; Narváez-Tovar C. A.; Garcia-Bravo J. M. Characterization of 3D-printed lenses and diffraction gratings made by DLP additive manufacturing. Rapid Prototyp. J. 2019, 25, 1684–1694. 10.1108/RPJ-03-2019-0074.

[ref13] Yuan C.; Kowsari K.; Panjwani S.; Chen Z.; Wang D.; Zhang B.; Ng C. J.-X. Ultrafast Three-Dimensional Printing of Optically Smooth Microlens Arrays by Oscillation-Assisted Digital Light Processing. ACS Appl. Mater. Interfaces 2019, 11, 40662–40668. 10.1021/acsami.9b14692. 31589018

[ref14] Luo Y.; Canning J.; Zhang J.; Peng G.-D. Toward optical fibre fabrication using 3D printing technology. Opt. Fiber Technol. 2020, 58, 102299 10.1016/j.yofte.2020.102299.

[ref15] Robles-Martinez P.; Xu X.; Trenfield S. J.; Awad A.; Goyanes A.; Telford R.; Basit A. W.; Gaisford S. 3D printing of a multi-layered polypill containing six drugs using a novel stereolithographic method. Pharmaceutics 2019, 11, 274 10.3390/pharmaceutics11060274. 31212649 PMC6630370

[ref16] Zhang B.; Li S.; Hingorani H.; Serjouei A.; Larush L.; Pawar A. A.; Goh W. H.; Sakhaei A. H.; Hashimoto M.; Kowsari K.; Magdassi S.; Ge Q. Highly stretchable hydrogels for UV curing based high-resolution multimaterial 3D printing. J. Mater. Chem. B 2018, 6, 3246–3253. 10.1039/c8tb00673c. 32254382

[ref17] Kowsari K.; Akbari S.; Wang D.; Fang N. X.; Ge Q. High-efficiency high-resolution multimaterial fabrication for digital light processing-based three-dimensional printing. 3D Print. Addit. Manuf. 2018, 5, 185–193. 10.1089/3dp.2018.0004.

[ref18] Gong P.; Li Y.; Xin C.; Chen Q.; Hao L.; Sun Q.; Li Z. Multimaterial 3D-printing barium titanate/carbonyl iron composites with bilayer-gradient honeycomb structure for adjustable broadband microwave absorption. Ceram. Int. 2022, 48, 9873–9881. 10.1016/j.ceramint.2021.12.190.

[ref19] Joralmon D.; Alfarhan S.; Kim S.; Tang T.; Jin K.; Li X. Three-Dimensional Printing of Liquid Crystals with Thermal Sensing Capability via Multimaterial Vat Photopolymerization. ACS Appl Polym Mater. 2022, 4, 2951–2959. 10.1021/acsapm.2c00322.

[ref20] Chartrain N. A.; Williams C. B.; Whittington A. R. A review on fabricating tissue scaffolds using vat photopolymerization. Acta Biomater. 2018, 74, 90–111. 10.1016/j.actbio.2018.05.010. 29753139

[ref21] Zhou L.-Y.; Fu J.; He Y. A Review of 3D Printing Technologies for Soft Polymer Materials. Adv. Funct. Mater. 2020, 30, 2000187 10.1002/adfm.202000187.

[ref22] Huang P.; Deng D.; Chen Y. Modeling and fabrication of heterogeneous three-dimensional objects based on additive manufacturing. In ASME International Mechanical Engineering Congress and Exposition; American Society of Mechanical Engineers, 2013: p. V02AT02A056.

[ref23] Matte C.-D.; Pearson M.; Trottier-Cournoyer F.; Dafoe A.; Kwok T.-H. Multi-material digital light processing printer with material tower and spray cleaning. In International Manufacturing Science and Engineering Conference; American Society of Mechanical Engineers, 2018; p. V004T03A063.

[ref24] Nair S. S.; Heinrich A.; Klein M.; Steenhusen S. Additive manufacturing of photoluminescent optics; Proceedings Organic Photonic Materials and Devices XXI; SPIE, 2019: p. 1091505.

[ref25] Rank M.; Sigel A.; Bauckhage Y.; Suresh-Nair S.; Dohmen M.; Eder C.; Berge C.; Heinrich A. 3D Printing of Optics Based on Conventional Printing Technologies. In: 3D Printing of Optical Components; A., Heinrich, Ed.; Springer International Publishing: Cham, 2020: pp 45–167.

[ref26] Han D.; Yang C.; Fang N. X.; Lee H. Rapid multi-material 3D printing with projection micro-stereolithography using dynamic fluidic control. Addit. Manuf. 2019, 27, 606–615. 10.1016/j.addma.2019.03.031.

[ref27] Han L.-H.; Suri S.; Schmidt C. E.; Chen S. Fabrication of three-dimensional scaffolds for heterogeneous tissue engineering. Biomed. Microdevices 2010, 12, 721–725. 10.1007/s10544-010-9425-2. 20393801

[ref28] Hisham M.; Saravana Kumar G.; Deshpande A. P. Process optimization and optimal tolerancing to improve dimensional accuracy of vat-photopolymerized functionally graded hydrogels. Results Eng. 2022, 14, 100442 10.1016/j.rineng.2022.100442.

[ref29] Li Z.; Liu P.; Ji X.; Gong J.; Hu Y.; Wu W.; Wang X.; Peng H. Q.; Kwok R. T. K.; Lam J. W. Y.; Lu J.; Tang B. Z. Bioinspired Simultaneous Changes in Fluorescence Color, Brightness, and Shape of Hydrogels Enabled by AIEgens. Adv. Mater. 2020, 32, 1906493 10.1002/adma.201906493. 32022969

[ref30] Iezzi B.; Afkhami Z.; Sanvordenker S.; Hoelzle D.; Barton K.; Shtein M. Electrohydrodynamic Jet Printing of 1D Photonic Crystals: Part II—Optical Design and Reflectance Characteristics. Adv. Mater. Technol. 2020, 5, 2000431 10.1002/admt.202000431.

[ref31] Benjamin A. D.; Abbasi R.; Owens M.; Olsen R. J.; Walsh D. J.; LeFevre T. B.; Wilking J. N. Light-based 3D printing of hydrogels with high-resolution channels. Biomed. Phys. Eng. Express. 2019, 5, 025035 10.1088/2057-1976/aad667.

[ref32] O’Neill P. F.; Kent N.; Brabazon D. Mitigation and control of the overcuring effect in mask projection micro-stereolithography. AIP Conf. Proc. 2017, 1896, 200012 10.1063/1.5008249.

[ref33] Alamoudi N. B.; AlShammari R. Z.; AlOmar R. S.; AlShamlan N. A.; Alqahtani A. A.; AlAmer N. A. Prevalence of color vision deficiency in medical students at a Saudi University. J. Family Community Med. 2021, 28, 196 10.4103/jfcm.jfcm_235_21. 34703380 PMC8496699

[ref34] Salih A. E.; Shanti A.; Elsherif M.; Alam F.; Lee S.; Polychronopoulou K.; Almaskari F.; AlSafar H.; Yetisen A. K.; Butt H. Silver Nanoparticle-Loaded Contact Lenses for Blue-Yellow Color Vision Deficiency. Phys. Status Solidi A 2022, 219, 2100294 10.1002/pssa.202100294.

[ref35] Salih A. E.; Elsherif M.; Alam F.; Yetisen A. K.; Butt H. Gold Nanocomposite Contact Lenses for Color Blindness Management. ACS Nano 2021, 15, 4870–4880. 10.1021/acsnano.0c09657. 33570901 PMC8023801

[ref36] Salih A. E.; Elsherif M.; Alam F.; Alqattan B.; Yetisen A. K.; Butt H. Syntheses of Gold and Silver Nanocomposite Contact Lenses via Chemical Volumetric Modulation of Hydrogels. ACS Biomater. Sci. Eng. 2022, 2111 10.1021/acsbiomaterials.2c00174. 35468279 PMC9092337

[ref37] Hittini S.; Salih A. E.; Alam F.; Shanti A.; Lee S.; Polychronopoulou K.; AlSafar H.; Almaskari F.; Butt H. Fabrication of 3D-Printed Contact Lenses and Their Potential as Color Blindness Ocular Aids. Macromol. Mater. Eng. 2023, 10.1002/mame.202200601.

[ref38] Roostaei N.; Hamidi S. M. Two-dimensional biocompatible plasmonic contact lenses for color blindness correction. Sci. Rep. 2022, 12, 2037 10.1038/s41598-022-06089-8. 35132172 PMC8821612

[ref39] Zhang F.; Kurokawa K.; Lassoued A.; Crowell J. A.; Miller D. T. Cone photoreceptor classification in the living human eye from photostimulation-induced phase dynamics. Proc. Natl. Acad. Sci. U. S. A. 2019, 116, 7951–7956. 10.1073/pnas.1816360116. 30944223 PMC6475411

[ref40] Schnapf J. L.; Kraft T. W.; Baylor D. A. Spectral sensitivity of human cone photoreceptors. Nature 1987, 325, 439–441. 10.1038/325439a0. 3808045

[ref41] Lin H.-Y.; Chen L.-Q.; Wang M.-L. Improving discrimination in color vision deficiency by image re-coloring. Sensors 2019, 19, 2250 10.3390/2Fs19102250. 31096676 PMC6567888

[ref42] Neitz J.; Neitz M. The genetics of normal and defective color vision. Vision Res. 2011, 51, 633–651. 10.1016/j.visres.2010.12.002. 21167193 PMC3075382

[ref43] Salih A. E.; Elsherif M.; Ali M.; Vahdati N.; Yetisen A. K.; Butt H. Ophthalmic Wearable Devices for Color Blindness Management. Adv. Mater. Technol. 2020, 5, 1901134 10.1002/admt.201901134.

[ref44] el Moussawi Z.; Boueiri M.; Al-Haddad C. Gene therapy in color vision deficiency: a review. Int. Ophthalmol. 2021, 41, 1917–1927. 10.1007/s10792-021-01717-0. 33528822

[ref45] Oli A.; Joshi D. Efficacy of red contact lens in improving color vision test performance based on Ishihara, Farnsworth D15, and Martin Lantern Test. Med. J. Armed Forces India. 2019, 75, 458–463. 10.1016/j.mjafi.2018.08.005. 31719742 PMC6838496

[ref46] Sekar P.; Dixon P. J.; Chauhan A. Pigmented contact lenses for managing ocular disorders. Int. J. Pharm. 2019, 555, 184–197. 10.1016/j.ijpharm.2018.11.052. 30465853

[ref47] Alam F.; Elsherif M.; AlQattan B.; Ali M.; Ahmed I. M. G.; Salih A.; Antonysamy D. S.; Yetisen A. K.; Park S.; Butt H. Prospects for Additive Manufacturing in Contact Lens Devices. Adv. Eng. Mater. 2021, 23, 2000941 10.1002/adem.202000941.

[ref48] Alam F.; Elsherif M.; Alqattan B.; Salih A.; Lee S. M.; Yetisen A. K.; Park S.; Butt H. 3D Printed Contact Lenses. ACS Biomater. Sci. Eng. 2021, 7, 794–803. 10.1021/acsbiomaterials.0c01470. 33464813 PMC8396802

