# Supplementary material for: 3D Printing of Multimaterial Contact Lenses
Source: ACS Biomater Sci Eng. 2023 Jun 26;9(7):4381–91. doi: 10.1021/acsbiomaterials.3c00175 (PMC10336843; doi:10.1021/acsbiomaterials.3c00175)
Supplement: Supplementary file 1 — ab3c00175_si_001.pdf [file ab3c00175_si_001.pdf]

Supporting Information

3D Printing of Multimaterial Contact Lenses

Muhammed Hisham[a], Ahmed E. Salih[a], Haider Butt[a*]

[a] Department of Mechanical Engineering, Khalifa University, Abu Dhabi, 127788, UAE

**Corresponding Author: haider.butt@ku.ac.ae;*

SI Content

Number of pages: 8

Number of figures: 8

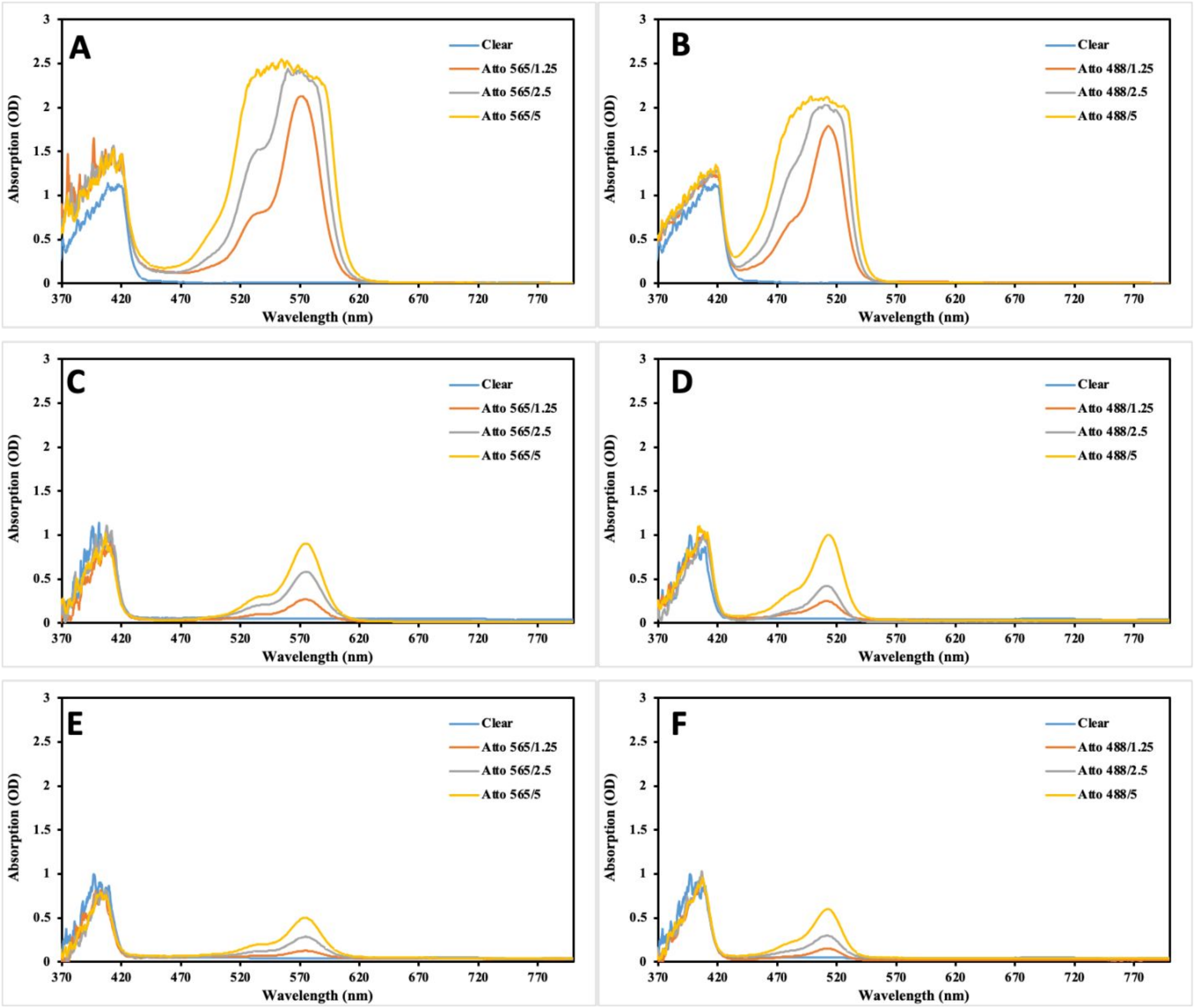

Figure S1. Absorption spectra from liquid resin with (a) Atto565, and (b) Atto488. Absorption spectra of 3D printed disks having (c) Atto565 and thickness 1mm, (d) Atto488 and thickness 1mm, (e) Atto565 and thickness 0.5mm, and (d) Atto488 and thickness 0.5mm.

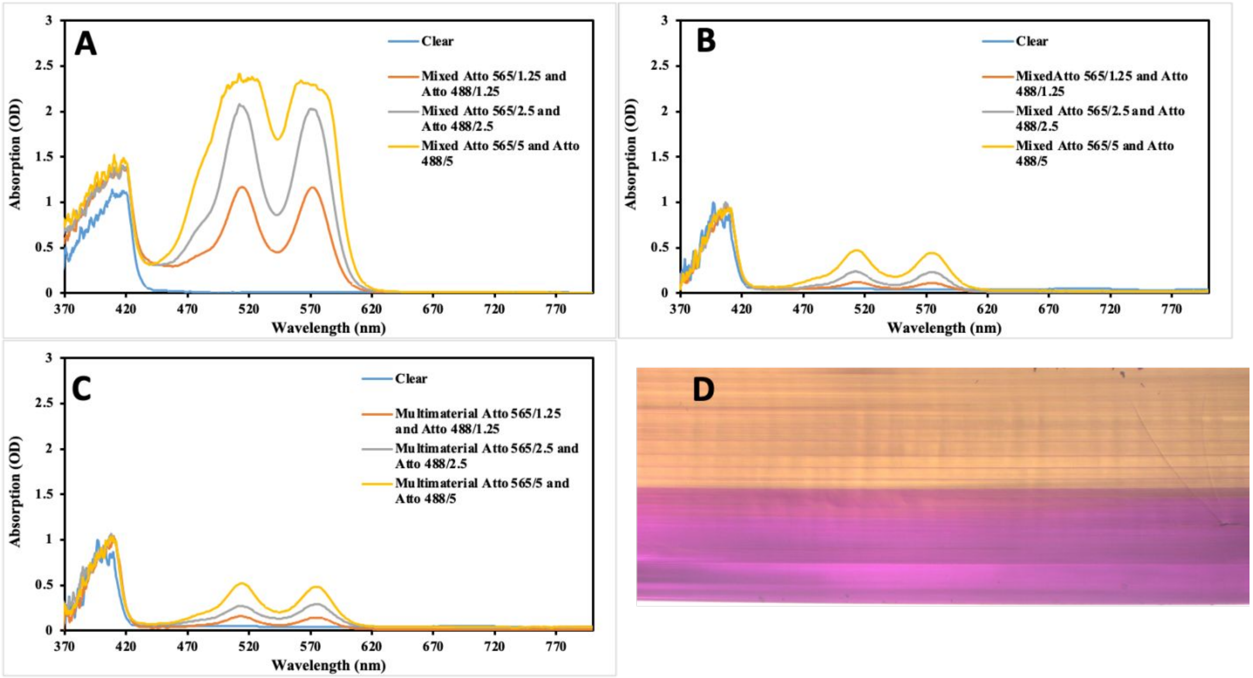

Figure S2. (a) Absorption spectra of liquid resin with both Atto565 and Atto488. Absorption spectra of 3D printed disks (1mm thick) made of (b) Atto565 and Atto488 mixed together, and (c) multimaterial samples having Atto565 and Atto488 in separate sections. (c) Cross-section of a printed disk made of multimaterial Atto565 and Atto488, both of concentration 2.5%.

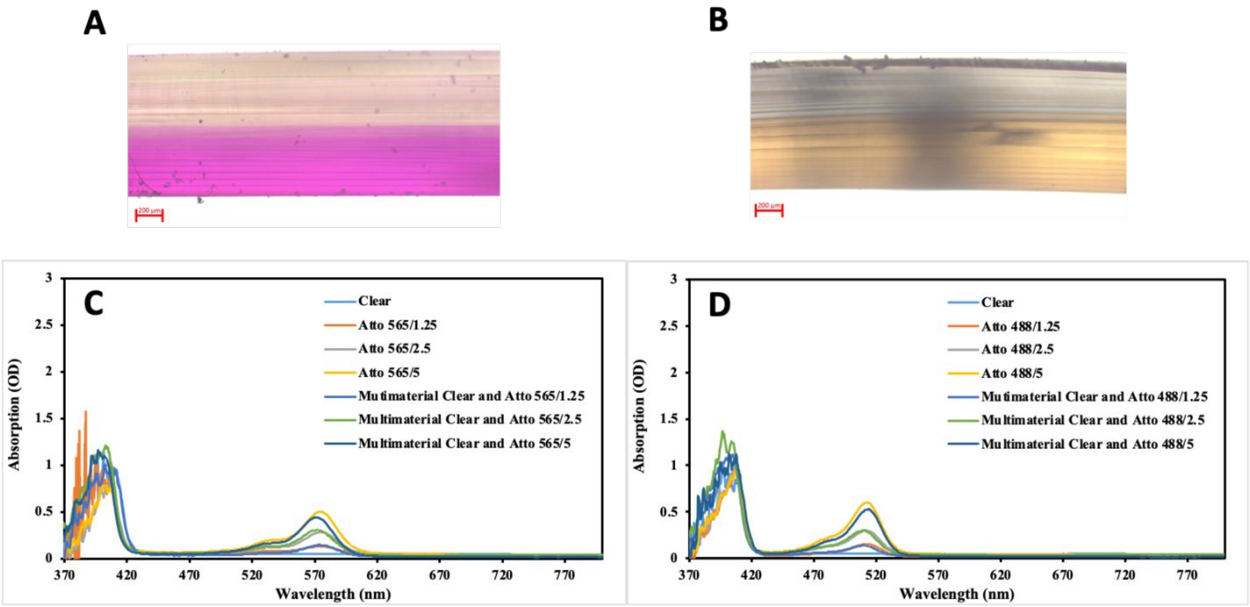

Figure S3. Cross-sections of (a) clear:Atto565 and (b) clear:Atto488 disks having concentration 2.5%. Comparison of transmission spectra from 3D printed multimaterial disks having thickness 1mm and single-material disks having thickness 0.5mm, for (c) clear:Atto565, and (d) clear:Atto488.

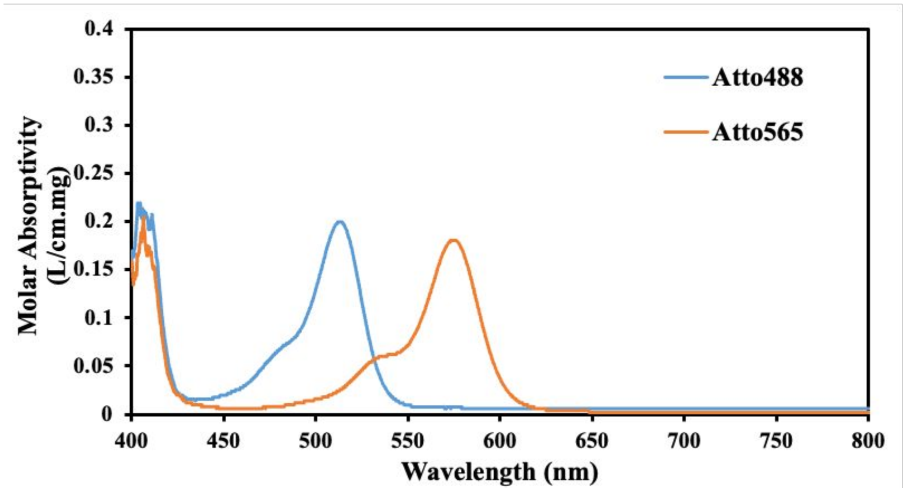

Figure S4. Molar absorptivity of Atto565 and Atto488 dyes with respect to wavelength, calculated from the absorbance spectrum of 3D printed samples. The molar absorptivity was calculated using the equation, Molar Absorptivity (L/cm.mg) = (Concentration (mg/L) x Thickness (cm)) / Absorbance.

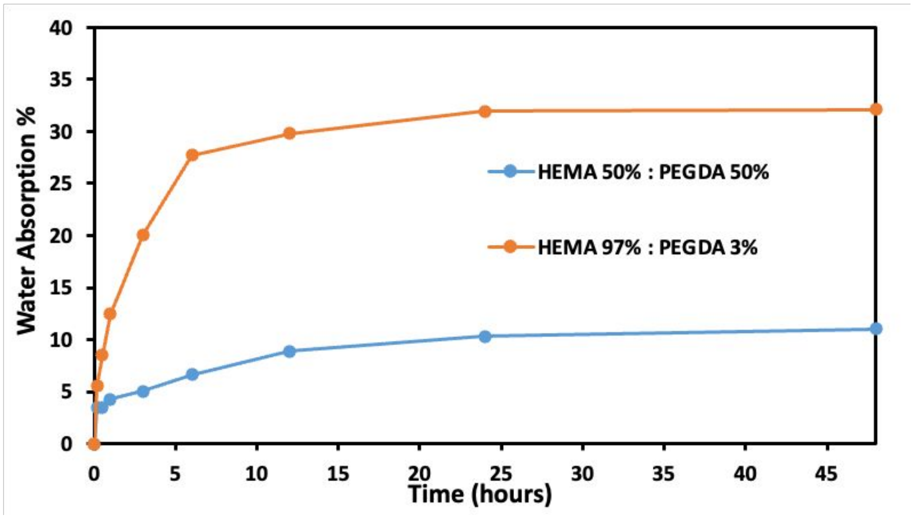

Figure S5. Water absorption with time for 3D printed samples with two different HEMA:PEGDA concentrations.

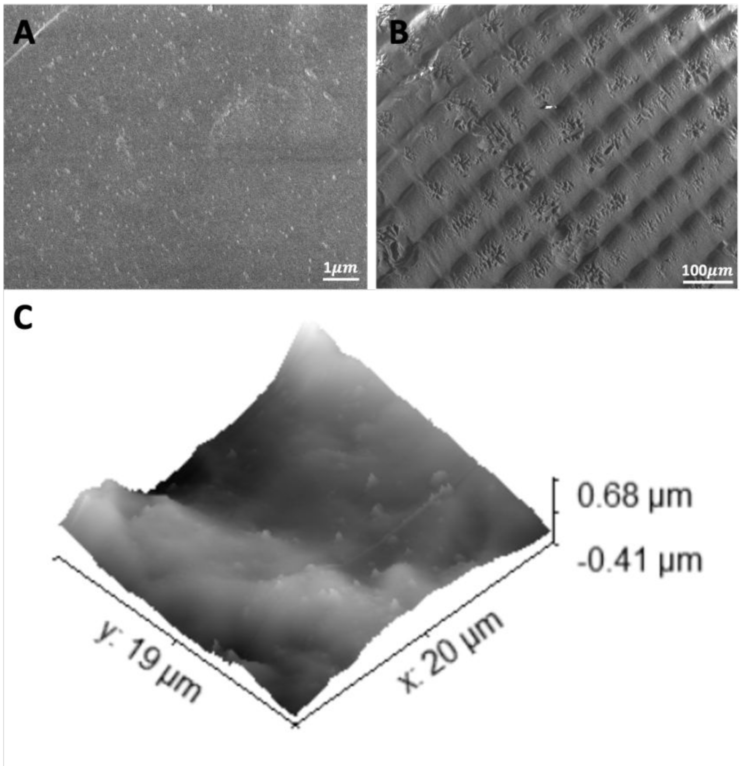

Figure S6. SEM images of the surface for 3D printed samples: (a) disc and (b) contact lens. (c) AFM 3D surface profile for 3D printed contact lens.

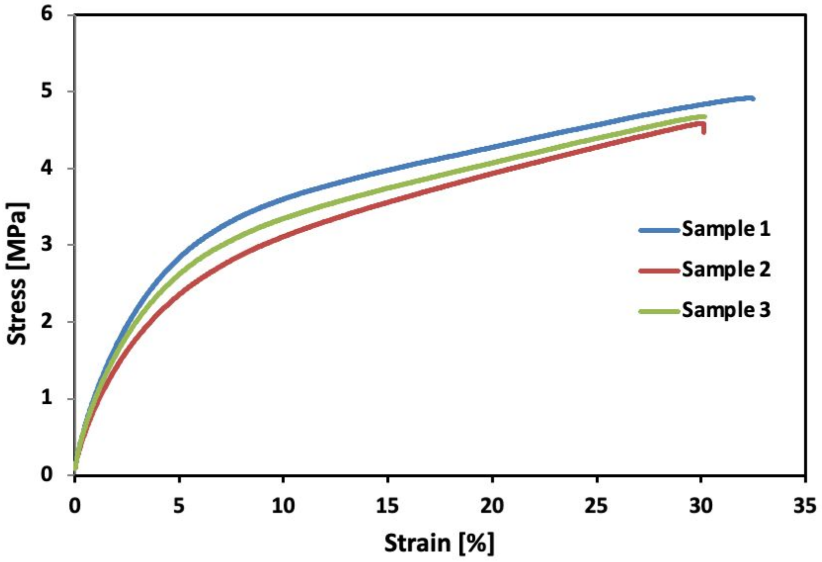

Figure S7. Tensile behavior of 3D printed samples printed with the HEMA/PEGDA resin used in this study. The test was performed with ASTM D638 (type IV) samples tested on Zwick-Roell Z005 UTM fitted with a 2.5 kN load cell.

Cell Viability Study

An MTT reduction assay (with (3-(4,5-Dimethylthiazol-2-yl)-2,5-diphenyltetrazolium bromide) was used to determine the cytotoxicity of 3D printed clear HEMA/PEGDA hydrogel lenses. Human dermal fibroblast cells were cultured in Dulbecco's Modified Eagle Medium, incubated at 37°C and 5% CO_2, and post-treated with dimethyl sulfoxide (DMSO). By comparing the absorbance of the control cells (cells with medium alone and no hydrogel) after 24 hours to that of the cells with the hydrogel, the percentage cell viability was determined. Percentage cell viability is calculated as:

$$Percentage\ Cell\ Viability = \frac{Absorbance\ of\ cells\ with\ the\ hydrogel}{Absorbance\ of\ control\ cells\ without\ the\ hydrogel} x100$$

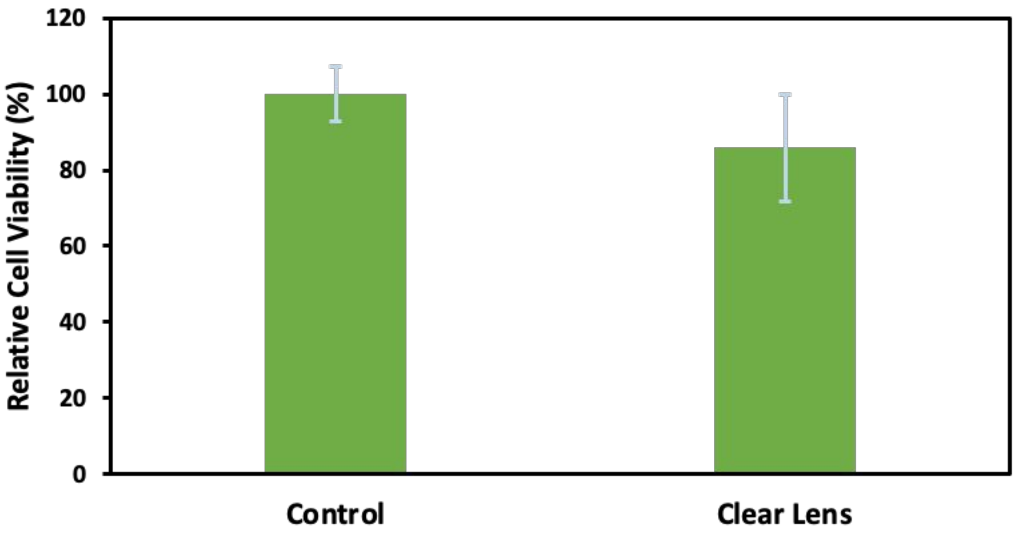

Figure S8. Relative cell viability for clear lenses 3D printed with HEMA/PEGDA resin.

The 3D printed hydrogel lenses displayed an average cell viability above 85%. ISO 10993-5:2009 stipulates that a substance is cytotoxic if it has a cell viability below 70%. Hence, the 3D printed HEMA/PEGDA lenses are determined to be non-cytotoxic in nature.

Cost Estimation

The cost of the contact lens includes material cost and 3D printing cost.

The material cost estimation is done as follows:
HEMA price - $ 0.05 per ml.
PEGDA price – $ 0.2 per ml.
TPO Price – $ 3.5 per gram.
Atto dye price - $ 100 for 1mg powder. (1mg is initially dissolved in 20ml resin stock)

Thus,
Cost of mixed resin = 0.5 x HEMA price + 0.5 x PEGDA price + 0.05 x TPO price
= 0.5 x 0.05 + 0.5 x 0.2 + 0.05 x 3.5
= $ 0.3 per ml

Cost of resin when dye is added at 5% concentration = $ 0.3 + 0.05x (1/20) x 100
= $ 0.55 per ml

One contact lens has a volume around 0.2ml. Considering that 0.5ml resin is required for each lens, to account for material losses while transfer and cleaning.

Thus, material cost for one contact lens = 0.5ml x $ 0.55 per ml
= $ 0.28

We used Wanhao D8 DLP printer in this study. The print time for the contact lens is around 1 hour. However, around 60 contact lenses can be printed in simultaneously in this 1 hour.

Wanhao D8 3D printer costs $999.
Considering that the printer is used for 2 years, this will imply a cost of $999/(2 x 365 x 24) = $0.057/hour.

Considering an electricity consumption of 250W and an electricity cost of $0.2/KWh,
We get the electricity cost = $0.2 x (250/1000) = $0.05/hour.

Thus, the total cost for 3D printing is $ 0.057 + 0.05 = $0.107/hour.
We can approximate this as $0.3/hour to include other additional costs like operator cost and maintenance.

For 60 contact lenses printed with a print time of 1 hour,
The 3D printing cost is = ($0.3/hour x 1 hour)/60 = $0.005 per contact lens.

Thus,
The net cost for one contact lens is = 3D printing cost + Material cost
= $0.005 + $ 0.28
= $0.285
≈ $0.29
